# circPSMC3: ceRNA and tumor suppressor

**DOI:** 10.18632/oncotarget.26953

**Published:** 2019-05-28

**Authors:** Chen Lu, Dawei Rong, Weiwei Tang

**Affiliations:** Department of General Surgery, Nanjing First Hospital, Nanjing Medical University, Nanjing, Jiangsu, China

**Keywords:** circRNAs, gastric cancer, sponge, biomarker

Gastric cancer (GC) is the third leading cause of cancer-related death worldwide and is a major cause of cancer-related mortality in China [1]. Since carcinogenesis in GC is a complex process, its etiology, genetic and epigenetic changes have been widely studied. Previous studies have shown that some genetic abnormalities, such as abnormal genes, microRNAs (miRNAs), long non-coding RNAs (lncRNAs) and circRNAs (circRNAs) are involved in the occurrence and development of GC [2–3], but the contribution of pathogenic mechanisms, especially emerging circRNAs, remains to be elucidated.

Emerging evidence indicates that the mechanisms of circRNAs mainly include I) function as competing endogenous RNAs or miRNA sponges [4]; II) regulating alternative splicing or transcription [5]; III) interacting with RNA-binding proteins (RBPs) [6]; IV) few circRNAs can be translated [7]. Current researches focus on how circRNAs that work as miRNA sponges to participate in the occurrence and development of carcinomas. Recently our group has confirmed that a new circRNA named circPSMC3 rather than liner PSMC3 mRNA was down-regulated in GC tissues, corresponding plasmas from GC patients as well as GC cell lines compared to normal controls. Lower circPSMC3 expression in GC patients was correlated with higher TNM stage and shorter overall survival. Importantly, we demonstrated that circPSMC3 could act as a sponge of miR-296-5p to regulate the expression of Phosphatase and Tensin Homolog (PTEN), and further suppress the tumorigenesis of gastric cancer cells (Figure 1) [8]. This is not the first article to study the role of cirRNAs in gastric cancer. We simply counted the number of articles on the role of cirRNAs in gastric cancer in the past 6 years, and found that the research volume has increased year by year. We hope more scholars will explore the mechanism and pathophysiological changes of cirRNAs in the progression of gastric cancer in the future.

**Figure 1:**
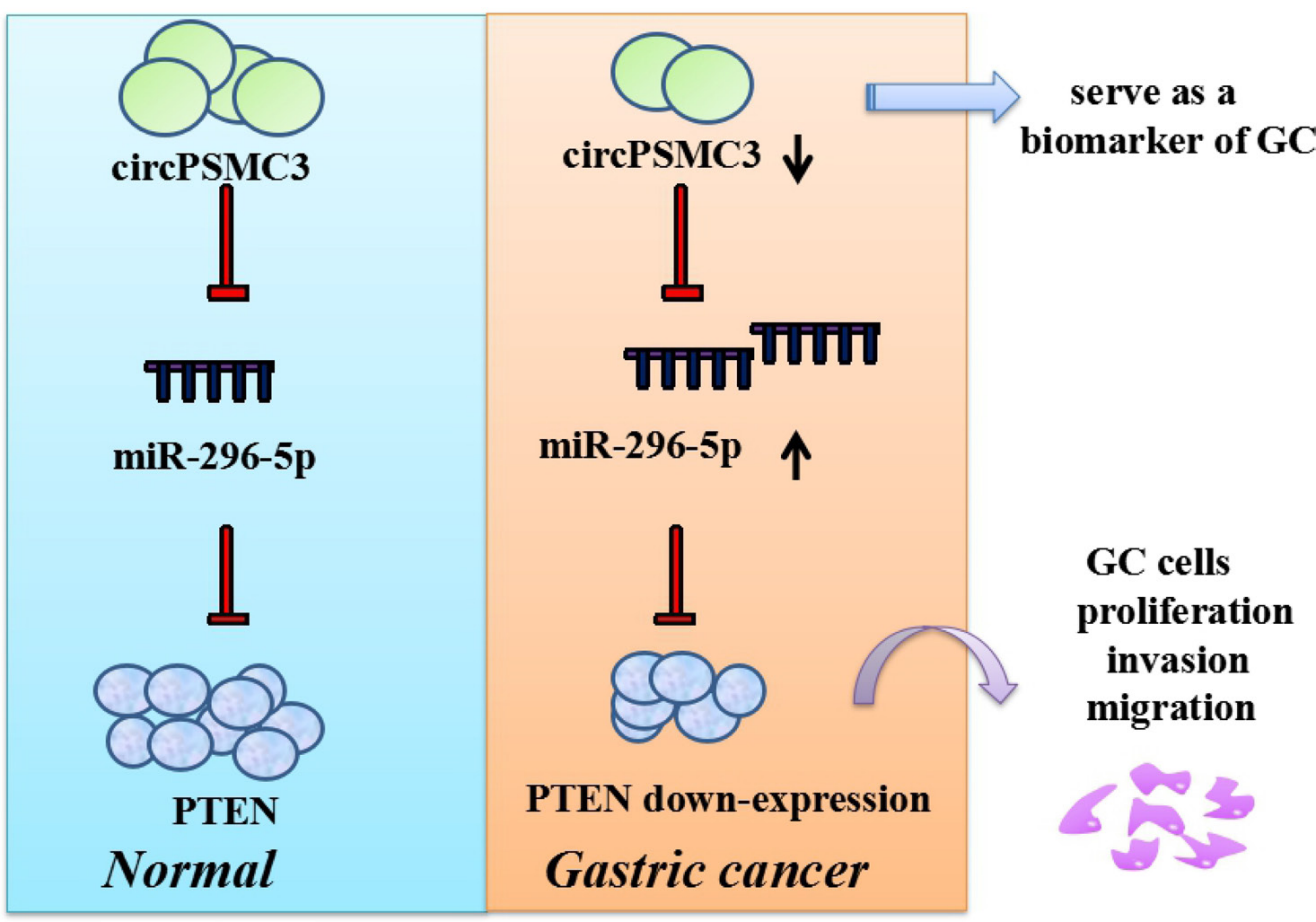
The schematic diagram of circPSMC3. circPSMC3 could serve as a circulating biomarker in GC and act as a sponge of miR-296-5p to regulate the expression of PTEN, further suppressing the tumorigenesis of gastric cancer cells.

The biggest highlight of our research is that the circRNA expression signatures in gastric cancer plasma were explored by using circRNA microarray analysis using plasma samples from 10 GC patients, including 5 patients with no lymph node metastasis and the other 5 patients with lymph node metastasis, and 5 normal individuals. We demonstrate that circPSMC3 can be used as a circulating biomarker in GC, which has been shown to have resistance to RNase R digestion based on the high stability of circRNAS in plasma of GC patients.

So can circPSMC3 be used as a therapeutic target for gastric cancer? Some factors should be taken into consideration for designing circRNAs as therapeutic targets: the general drug target expression abundance is high, and circPSMC3 expression level is relatively low in GC, so the target may be difficult to achieve at present. In addition, circRNAs may prove useful as predictive markers for chemotherapy sensitivity in tailored anticancer treatment [9]. Whether circPSMC3 is involved in chemotherapy resistance of gastric cancer remains to be further revealed.

## CONFLICTS OF INTEREST

The authors declare that they have no competing interests.
